# Immuno-scanning electron microscopy of islet primary cilia

**DOI:** 10.1242/jcs.262038

**Published:** 2024-05-28

**Authors:** Sanja Sviben, Alexander J. Polino, Isabella L. Melena, Jing W. Hughes

**Affiliations:** ^1^ Washington University Center for Cellular Imaging, Washington University School of Medicine, 660 South Euclid Ave, Saint Louis, MO 63110, USA; ^2^ Department of Cell Biology and Physiology, Washington University School of Medicine, 660 South Euclid Ave, Saint Louis, MO 63110, USA; ^3^ Department of Medicine, Washington University School of Medicine, 660 South Euclid Ave, Saint Louis, MO 63110, USA

**Keywords:** Immuno-SEM, Pancreatic islet, Primary cilia, Axoneme, Dynein

## Abstract

The definitive demonstration of protein localization on primary cilia has been a challenge for cilia biologists. Primary cilia are solitary thread-like projections that have a specialized protein composition, but as the ciliary structure overlays the cell membrane and other cell parts, the identity of ciliary proteins are difficult to ascertain by conventional imaging approaches like immunofluorescence microscopy. Surface scanning electron microscopy combined with immunolabeling (immuno-SEM) bypasses some of these indeterminacies by unambiguously showing protein expression in the context of the three-dimensional ultrastructure of the cilium. Here, we apply immuno-SEM to specifically identify proteins on the primary cilia of mouse and human pancreatic islets, including post-translationally modified tubulin, intraflagellar transport (IFT)88, the small GTPase Arl13b, as well as subunits of axonemal dynein. Key parameters in sample preparation, immunolabeling and imaging acquisition are discussed to facilitate similar studies by others in the cilia research community.

## Teaser

**Summary:** Combining scanning electron microscopy with immunogold labeling is used to identify proteins on primary cilia in human and mouse pancreatic islets.

## INTRODUCTION

Primary cilia are surface sensory organelles that perform vital functions for the cell. The structure of the primary cilium has been studied for many years by transmission electron microscopy (TEM), which has driven most of our understanding and classification of primary versus motile cilia. However, it has become clear from recent three-dimensional (3D) ultrastructural studies (Müller et al., 2020, 2024 preprint; Sun et al., 2019; Xu et al., 2021) that in pancreatic islets, the microtubule arrangements of primary cilia can deviate from their classic ‘9+0’ scheme, and that functionally primary cilia are not static organelles as once thought but can rather be dynamic and exhibit motility (Battle et al., 2015; Cho et al., 2022; Nonami et al., 2013; Ott and Lippincott-Schwartz, 2012). Elucidating the functional significance of these cilia properties requires identification of mechanistic protein components. This has so far been done by conventional immunofluorescence (IF) imaging, which has allowed localization of axonemal dynein and motility-related proteins to human islet primary cilia (Cho et al., 2022). However, there are pitfalls in IF colocalization studies that limit interpretation, a key one being the physical overlap among the cilium, plasma membrane and other cell parts that confound true colocalization, and that light microscopy generally lacks the resolution to show sub-ciliary protein expression. As result, there has not been a good method to unambiguously identify individual proteins and their 3D distribution on primary cilia.

Scanning electron microscopy (SEM) is a high-resolution technique uniquely suited for examining the native 3D conformation of surface structures, an approach that we recently used to characterize the primary cilia axoneme of human islets (Polino et al., 2023). In the present study, we combine our SEM protocol with antibody staining (immuno-SEM) to identify specific ciliary protein components. Using 18 nm colloidal gold-conjugated secondary antibodies, we obtain clear labeling of axonemal proteins *in situ* and demonstrate adaptability of this technique across species in mouse and human islets.

## RESULTS

We screened seven primary antibodies for cilia immuno-SEM in mouse and human islets and achieved compelling labeling with six out of seven. These primary antibodies were used in conjunction with standard gold-conjugated secondary antibodies specific for mouse and rabbit IgG. Table 1 lists the source and labeling conditions of all primary antibodies, where were typically used at 5- to 10-fold higher concentrations than for immunofluorescence imaging (Cho et al., 2022; Hughes et al., 2020; Li et al., 2022; Sanchez et al., 2022). Some but not all primary antibodies were tested in both mouse and human islets – species information is indicated in figures.

**Table 1.**
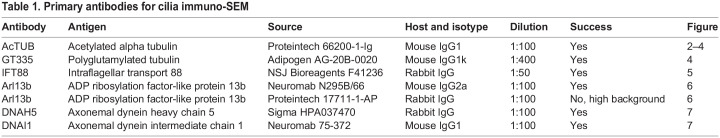
Primary antibodies for cilia immuno-SEM

Because immuno-SEM techniques had not been previously reported for primary cilia and because we anticipated difficulties in antibody labeling, we took several measures from the outset to enhance protocol performance. First, we opted to include a membrane removal (demembranating) step in our islet sample preparation because we reasoned that membrane stripping would expose axonemal antigens and increase the success of antibody labeling. Second, we included a gentle pre-fixation step using 0.25% glutaraldehyde (GA) prior to demembranation, which we thought would be crucial for crosslinking axoneme-associated and juxta-membrane ciliary proteins, such as intraflagellar transport 88 (IFT88) and the small GTPase ADP ribosylation factor-like protein 13b (Arl13b). Although a small amount of GA was expected to help preserve ultrastructural integrity, we refrained from using concentrations greater than 0.25% GA so as to protect downstream immunolabeling. Third, we included a glycine quenching step during antibody binding to reduce sample autofluorescence and to enhance antibody-dependent signals (Rosas-Arellano et al., 2016). After antibody labeling, which was performed with a conventional 4% paraformaldehyde (PFA) fixation, islets underwent a final crosslink in high-concentration 2% GA before alcohol dehydration, critical point drying and SEM imaging. We applied this workflow to both mouse and human islet samples, and found it to be robust and reliable for most antibodies tested.

**Fig. 1.**
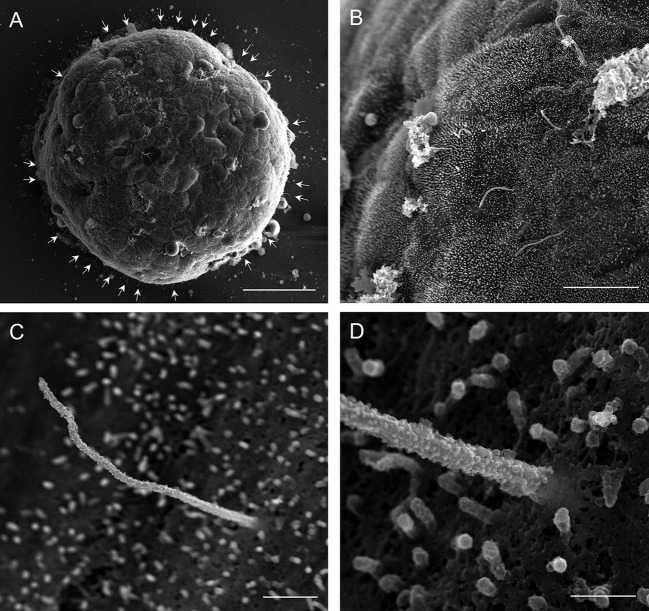
**Multi-scale SEM of islet primary cilia.** (A) An intact, demembranated mouse pancreatic islet scanned using an Everhart–Thornley detector (ETD). Small, thread-like primary cilia project from the islet surface (arrowheads). Taken at magnification 1200×. Scale bar: 50 μm. (B) Representative human islet surface scan using a through lens detector (TLD) showing dense short microvilli, solitary primary cilia and post-demembranation debris. Taken at magnification 4772×. Scale bar: 10 μm. (C) A human islet primary cilium imaged using a TLD detector. Taken at magnification 20,000×. Scale bar: 1 μm. (D) High-resolution scan of the cilium base from C using a TLD detector, Taken at magnification 65,000×. Scale bar: 400 nm. Images are representative of three independent experiments with 5–10 islets imaged per condition.

Multi-scale scans were acquired on intact islets adhered to glass coverslips, starting with whole-islet views at micrometer resolution and progressing to successive higher magnifications to visualize individual cilia and sub-ciliary zones at nanoscale resolution (Fig. 1). Cilia across spatial scales were tracked by morphology and their identification was aided by immunogold labeling of ciliary protein markers. Preservation of ciliary structures was excellent, as most cilia axonemes remained intact, exhibiting similar lengths (4–10 µm) and diameters (200–300 nm at base) to those previously characterized by us and others (Li et al., 2022; Nachury, 2014; Polino et al., 2023; Reiter et al., 2012). We found moderate magnification (20,000–35,000×) to give a good overview of the entire ciliary structure. In addition to primary cilia, other structures including microvilli and cortical cytoskeleton were also well-preserved on the islet surface.

**Fig. 2.**
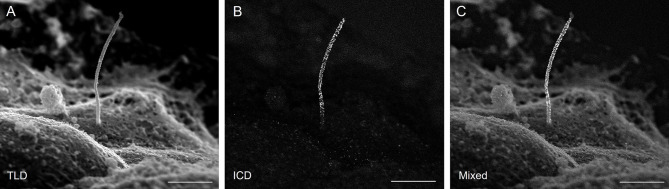
**Multi-detector scanning.** Simultaneous capture of secondary and backscatter electron images of a single mouse islet primary cilium labeled with anti-acetylated α-tubulin (Proteintech 66200-1-Ig, mouse IgG1, 1:100), Taken at magnification 17,000×. (A) Secondary electron imaging with through lens detector (TLD). (B) Backscatter electron imaging using an in column detector (ICD). (C) Mixed image generated through digital combining of TLD and ICD images. Mixed images provide the best visualization of immunogold position against islet surface textures and are used for most figures in this report. Scale bars: 2 μm. Images are representative of >50 cilia imaged over three experiments.

**Fig. 3.**
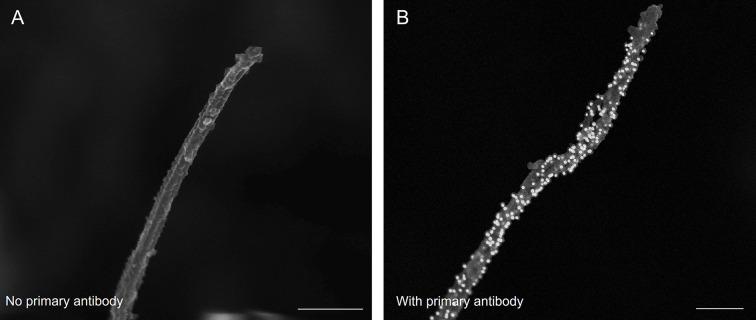
**Negative control for immuno-SEM.** Human islet cilia labeled (A) without or (B) with primary antibody to acetylated α-tubulin (Proteintech 66200-1-Ig, mouse IgG1, 1:100). The no-primary antibody condition controls for nonspecific binding of the secondary gold antibody (Jackson ImmunoResearch, donkey-anti-mouse #715-215-150, 1:20). Taken at magnification 65,000×. Scale bars: 400 nm. Images are representative of >20 cilia imaged in three experiments using human islets.

We acquired simultaneous images using multiple detectors. Fig. 2 shows images acquired with the TLD detector (which collects secondary electron signal for standard topography imaging), ICD detector (which collects backscattered electron signal that reveals immunogold label) and both detectors (overlayed TLD and ICD images; denoted Mixed). Gold particles are seen as white dots in the ICD image and overlap with cilia shape outline, confirming that imaging conditions were good and that labeling is detected. We validated the size of the gold dots by measuring bead diameter in ImageJ according to the pixel:length relationship set by the imaging scale. All gold dots measured 17–18 nm in diameter and had uniformly high electron density (mean gray value gold dots 52,000–54,000, cilia axoneme 20,000–21,000, background 4000–5000). Negative control was tested for both anti-mouse- and anti-rabbit-IgG secondary antibodies, which produced no labeling (Fig. 3).

**Fig. 4.**
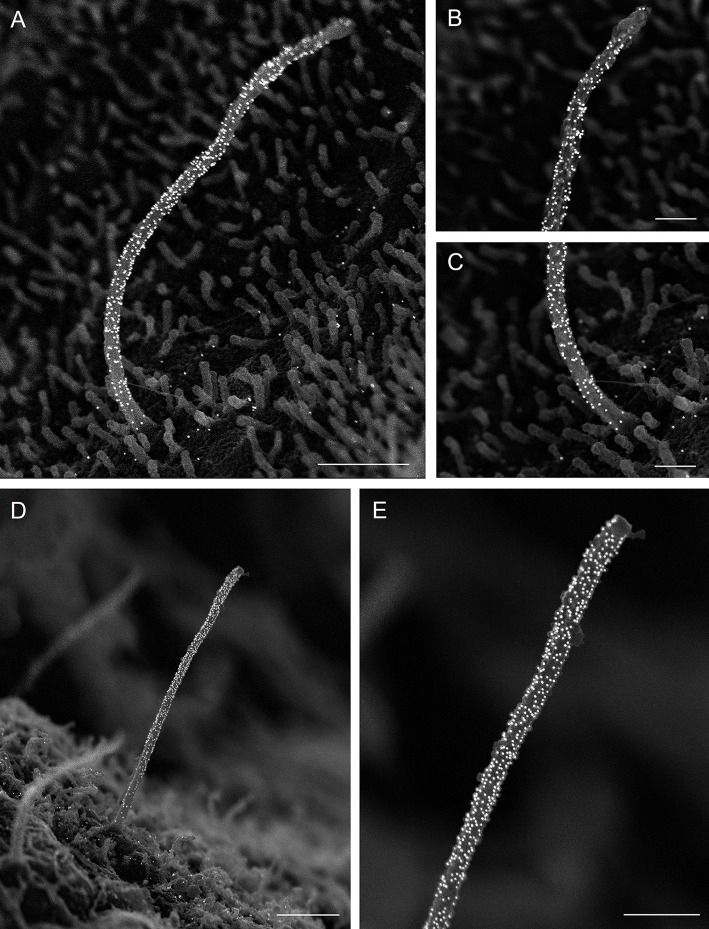
**Posttranslational modifications of tubulin in pancreatic islet cilia.** (A–C) Human islet primary cilium labeled with mouse-anti-acetylated α-tubulin (Proteintech 66200-1-Ig, mouse IgG1, 1:100), showing enrichment of gold particles on the ciliary axoneme, along with low background labeling on the cell surface. (A) Whole cilium. Taken at magnification 20,000×. Scale bar: 1 μm. (B) Cilium tip. Taken at magnification 65,000×. Scale bar: 400 nm. (C) Cilium base. Taken at magnification 20,000×. Scale bar: 400 nm. (D,E) Mouse islet cilia labeled by anti-polyglutamylated tubulin (AdipoGen GT335, mouse IgG1_k_ 1:400). (D) Whole cilium. Taken at magnification 20,000×. Scale bar: 1 μm. (E) Cilium tip. Taken at magnification 50,000×. Scale bar: 500 nm. Images are representative of three experiments.

**Fig. 5.**
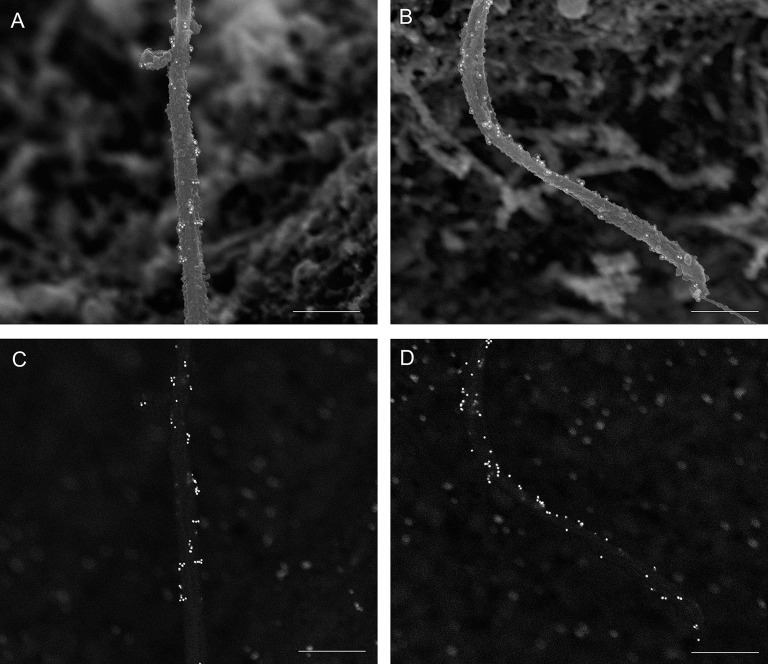
**IFT88 in mouse islet cilia.** Mixed ICD-TLD images (A,B) and corresponding single-detector ICD images (C,D) showing IFT88 labeling in mouse islets (rabbit-anti-IFT88, NSJ Bio F41236, 1:50). Taken at magnification 50,000×. Scale bars: 500 nm. Images are representative of 20 cilia from five mouse islets.

Antibody performance for immuno-SEM was comparable to their performance in IF imaging, with core axonemal tubulin being most easily detectable. Acetylated α-tubulin is a universal marker of primary cilia across tissue types and in our study reliably labeled the entire ciliary axoneme in mouse and human islets (Figs 2–4). A second post-translational modification of tubulin, polyglutamylated tubulin, was also readily detected through the entire length of the cilium (Fig. 4D,E). Intraflagellar transport IFT88 was detected in a scattered pattern along the entire axoneme, using an affinity-purified rabbit polyclonal antibody previously validated by IF (Sanchez et al., 2022) in islet primary cilia (Fig. 5). We did not observe discrete IFT train-like structures corresponding to IFT88 immunolabeling on the axonemal surface and reason that the train complexes might have been partially disrupted by the demembranation process. Other components of the axoneme might also have been disrupted, although the core microtubule bundle remained intact and we did not observe microtubule splaying as sometimes can be seen with EM specimen damage or structural mutations affecting ciliary microtubular integrity (Brody et al., 2024 preprint; Polino et al., 2023).

**Fig. 6.**
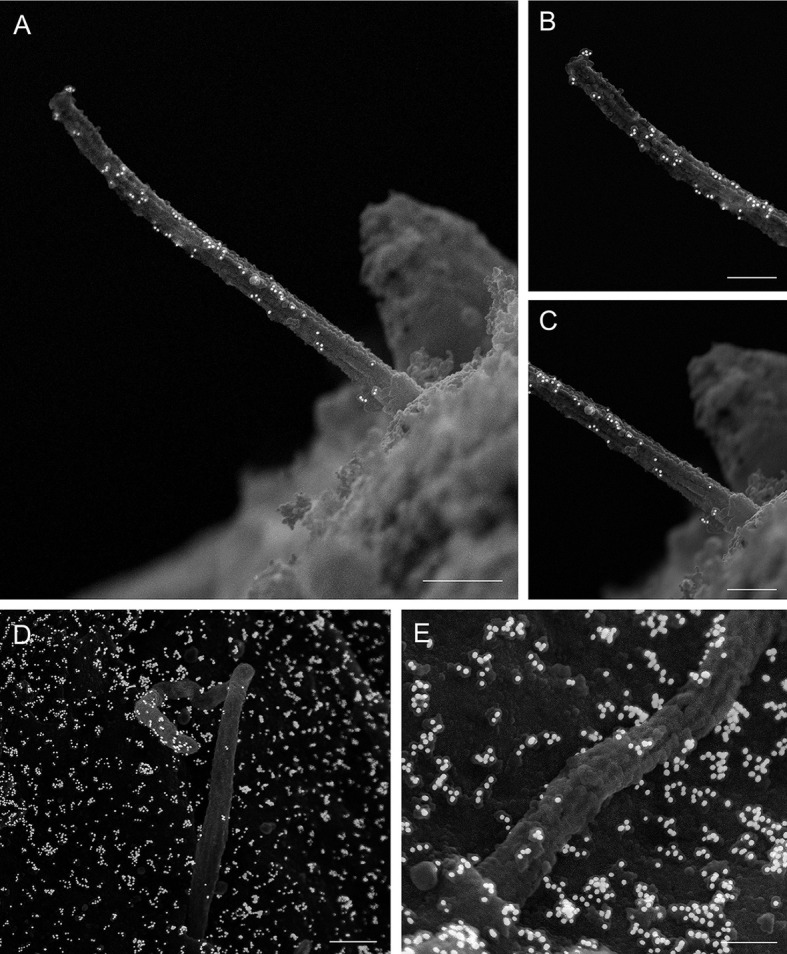
**Arl13b labeling in mouse islet cilia.** (A–C) Clean Arl13b immunolabeling is achieved using a monoclonal primary antibody (NeuroMab N295B/66, mouse IgG2a, 1:100). (A) Whole cilium. Taken at magnification 32,500×. Scale bar: 1 μm. (B,C) Distal cilium and tip. Taken at magnification 75,000×. Scale bar: 500 nm. (D,E) Another Arl13b antibody (Proteintech 17711-1-AP, rabbit polyclonal, 1:100) produced heavy background labeling on the islet surface, making it difficult to interpret ciliary signals. (D) Whole cilium. Taken at magnification 35,000×. Scale bar: 500 nm. (E) Cilia base. Taken at magnification 100,000×. Scale bar: 200 nm. Images are representative of >20 cilia per Arl13b antibody staining.

We did observe variable performance among antibodies for the same antigen, even those validated across other immunolabeling protocols. Arl13b is a small ciliary G-protein of the Ras superfamily and is a standard ciliary protein marker for IF studies. We tested two commercial Arl13b antibodies that our laboratory relies on for IF imaging. One antibody (Neuromab N295B/66, 1:100 dilution) gave clear ciliary labeling with near zero background signal on the membrane (Fig. 6A–C), whereas another antibody (Proteintech 17711-1-AP, 1:100 dilution) despite being a strong performer for IF gave such heavy background signal in immuno-SEM that rendered its ciliary labeling uninterpretable (Fig. 6D,E). This was a surprise given our experience of clean IF staining when using the Proteintech antibody, which highlights the need to empirically test each antibody for immuno-SEM despite prior validation for other imaging modalities.

**Fig. 7.**
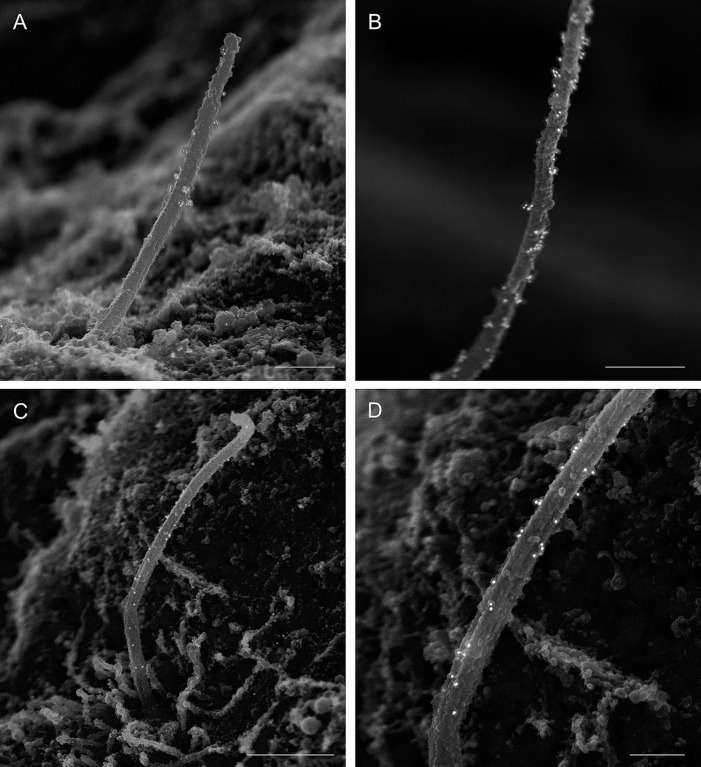
**Axonemal dynein in mouse islet cilia.** Presence of the motor dynein in the primary cilia axoneme as indicated by immunogold labeling of dynein heavy chain 5 (rabbit-anti-DNAH5, Sigma HPA 037470, 1:100) and dynein intermediate chain 1 (mouse-anti-DNAI1, NeuroMab 75-372, 1:100). (A) DNAH5, whole cilium. Taken at magnification 35,000×. Scale bar: 500 nm. (B) DNAH5, distal cilium. Taken at magnification 50,000×. Scale bar: 500 nm. (C) DNAI1, whole cilium. Taken at magnification 25,000×, Scale bar: 1 μm. (D) DNAI1, mid-axoneme. Taken at magnification 60,000×. Scale bar: 400 nm. Images are representative of 40 cilia per axonemal dynein antibody staining.

As we and others have observed motile properties in primary cilia (Battle et al., 2015; Cho et al., 2022; Ott and Lippincott-Schwartz, 2012), we tested the presence of axonemal dynein in islets. Two axonemal dynein antibodies, dynein heavy chain 5 (DNAH5) and dynein intermediate chain 1 (DNAI1), produced specific labeling on the ciliary axoneme in mouse islets (Fig. 7). This labeling is clean with low background but sparser than those of core axonemal markers such as acetylated or polyglutamylated tubulin, which might be attributable to either low antigen abundance or suboptimal antibody binding, or both. Nonetheless, the presence of multiple axonemal dynein components in mouse islets as demonstrated by immuno-SEM in the present study and by IF in human islets previously (Cho et al., 2022) suggests that islet primary cilia contain motor complexes and support the notion that primary cilia motility might be a conserved feature across species.

## DISCUSSION

Our study demonstrates that the immuno-SEM technique is suitable for protein identification in islet primary cilia using standard commercial antibodies. Whereas compelling protein localization data is often unattainable by immunofluorescence (IF) for reasons discussed in the introduction, we found immuno-SEM to offer the unique capability to demonstrate ciliary protein distribution while resolving 3D ultrastructural details. A key advance in our protocol is that, with demembranation, our method can be applied to labeling core axonemal proteins and is not limited to proteins on the ciliary surface. We discuss below a number of technical details key to the success and reproducibility of our immuno-SEM workflow.

### Sample stability and preservation

Sample detachment is a common problem for primary tissues such as islets, leading to attrition of imageable specimens throughout the workflow. This leads to increased time required for sample preparation and, often, means experiments to be repeated. In our experience, islets that were loosely attached were also unusable, as these had poor grounding and conductivity and therefore led to charging issues during imaging. For best success, we found that coating the glass coverslips with recombinant human laminin (Gibco rhLaminin-521) and allowing the islets to fully adhere, for up to 3 days, helped ensure that adequate islet numbers are preserved. Islet attachment and quality is routinely assessed throughout the workflow to ensure proper concentration and distribution on the coverslip.

### Fixation

Immuno-SEM is a hybrid protocol between immunohistochemistry and SEM, and therefore requires multiple chemical fixation steps dedicated for these different procedure segments. The choice of fixatives is important when considering antibody performance, just as it has been demonstrated for cilia IF imaging (Hua and Ferland, 2017). The requisite fixative for EM is glutaraldehyde (GA), which in high concentrations is incompatible with antibody labeling, yet must be included upfront to preserve ultrastructural morphology and at the end to crosslink primary–secondary antibody interaction. We found that islets could tolerate 0.25% glutaraldehyde (GA) during the demembranation step prior to antibody staining. We then rinse and fix the samples with 4% PFA as would be used for routine immunohistochemistry, and after antibody staining is complete, do a final fix with 2% GA for electron microscopy.

### Antibody performance

Antibody performance is essential to the success of immuno-SEM and depends on crucial factors, such as sample quality, antigen concentration and availability, fixative choice and labeling conditions. Generally, strong performance by an antibody in IF is a good indication that it will work for immuno-SEM. Still, this requires a certain amount of trial and error, as can be seen in our study, and optimization is needed to identify successful antibody dilution and staining conditions (Gulati et al., 2019). We found a number of steps to enhance primary antibody labeling: (1) glycine quenching, which increases signal specificity by reducing non-specific free aldehyde binding to the antibody (Rosas-Arellano et al., 2016), (2) membrane-stripping, which exposes antigens below the ciliary surface, and (3) using sufficient antibody concentrations, usually five to ten times higher than for IF, which is required to survive extensive washing and use of multiple fixatives. Given limited samples, particularly of human islets, we did not test multiple conditions for primary antibody labeling duration and instead relied on prior experience with immuno-SEM in other tissues. Shorter incubations (1–2 h versus overnight) might in fact be advantageous for reducing damage to the cilia ultrastructure given the high salt concentration (150 nM NaCl) in the phosphate-buffered saline (PBS) antibody buffer. For secondary antibodies, we used commercial 18 nm colloidal gold-conjugated Ig to mouse and rabbit (Jackson ImmunoResearch) for 1.5 h and found these to produce robust and reliable labeling.

### Inclusion of controls

The inclusion of proper negative and positive controls is essential to the rigor of any imaging study, immuno-SEM included. Unlabeled samples such as no-primary antibody provide good controls against nonspecific labeling by secondary colloidal gold. Of note, this does not demonstrate specificity of primary antibody binding, which would ideally be done by pre-incubating the antibody with the antigen peptide, replacing the primary antibody with a nonimmune Ig of the same isotype, or by validating in gene knockout samples. For a positive control, the use of alternative targeted antibodies to the same antigen and especially from a different host species would be a good way to validate labeling results.

### Continuity of workflow

Although the sample adhering and immunolabeling steps offer flexibility on timing and duration, certain parts of the protocol should be performed without interruption, as otherwise they can lead to processing issues. We found that mouse islets were susceptible to damage if they had a longer pause between critical point drying (CPD) and coating, which would lead to severe charging issues and make cilia difficult to image in high-resolution mode. This likely stems from moisture accumulation and trapping under the coating, which could be minimized by coating samples immediately after CPD and storing them in a desiccator until imaging. Human islets typically behaved more stably and did not have as many processing issues, but had fewer cilia on their surface, thus also required care during the workflow to not reduce sampling number.

### Sample charging and coating

Electric charging is a common problem during imaging, an issue stemming from the production of excess secondary electrons resulting in the sample surface becoming positively charged. The plasma of secondary electrons that forms on the surface of the sample interferes with further interaction of the incident beam with the sample, which results in a charging effect and the inability to keep electron beam in focus. In our islet samples, we frequently needed to image multiple areas of the coverslip because of charge built-up in certain regions. To combat charging, we used three strategies. First, low levels of osmium in the sample prep, 0.5% instead of the usual 2% used for TEM and SEM – the latter would make samples better grounded but given the high atomic number of osmium would generate excess backscatter signal to interfere with the gold label. Second, carbon coating, which deposits a thin layer of conductive material to reduce charge build-up by grounding excess electrons. We used 12 nm carbon rather than heavier metals such as platinum and chromium (Goldberg and Fišerová, 2022) which would have generated too much backscatter, Finally, limiting the accelerating voltage during imaging, for example, no more than 5 kilovolts (kV), which is on the high end for biological samples but was the level necessary on our Helios FIB-SEM system for producing sufficient backscatter electron signal to see the gold labeling. Some of these technical adjustments led to small changes to the specimen itself, for example carbon coating adds 12 nm to the sample surface thereby slightly increasing their physical dimensions, which should be accounted for when extrapolating size information. Carbon coating also likely contributed to ‘gumming up’ of the sample surface and limited our ability to resolve axonemal details and visualize gaps between microtubules, a caveat to be kept in mind for image interpretation.

### General use of immuno-SEM to study cilia in different contexts

Based on the reproducibility of immunogold labeling between mouse and human islets and among sample preparations, we believe our immuno-SEM protocol to be potentially applicable to studying cilia in broader contexts, including primary cilia in non-islet cells, motile cilia in multi-ciliated cells, embryonic nodal cilia, stereocilia, and indeed any other surface organelle. The basic requirements for feasibility of this approach include: (1) the structure of interest is stably exposed on the cell surface or otherwise accessible by SEM, (2) the specimen can be affixed to an imaging surface, e.g. a glass coverslip and survive extensive fixation and washing steps, and (3) validated primary antibodies exist for the proteins of interest. Although we only performed single protein labeling here, multiplexed labeling can be done using different sizes of gold particles, e.g. 12 and 18 nm. Demembranation might help expose intracellular epitopes on the core axoneme, whereas transmembrane proteins might be better detected on native, membrane-intact specimens.

In summary, we show the feasibility of the immuno-SEM technique for identifying and visualizing primary cilia proteins on pancreatic islets. This workflow performs robustly for multiple protein antigens tested in our study and across two species, mouse and human. We emphasize the need to optimize labeling conditions in each antibody, cell and tissue type, rather than strict adherence to our protocol. We hope that discussion of the crucial parameters in sample preparation and imaging will facilitate future immuno-SEM studies to enable high-resolution protein identification in cilia and related cell structures.

## MATERIALS AND METHODS

### Islet preparation for SEM

Mouse islets were isolated from wild-type C57Bl/6J mice using collagenase digestion with a modified Lacy protocol (Lacy and Kostianovsky, 1967). Both female and male mice were used in this study. Isolated islets were rested overnight in islet medium [RPMI 1640 with 11 mM glucose (Sigma, G7021), 10% FBS (GenClone, 25-514H) and 1% penicillin-streptomycin (Sigma, P4333)] to allow recovery of surface morphology. All animal experiments were performed according to approved guidelines. Human islets from a 42-year-old healthy, non-diabetic, non-obese female donor were obtained from the Integrated Islet Distribution Program (IIDP), and were also washed and rested overnight before use for experiments. Human donor islets were exempt from IRB Human Studies Approval. Islets were plated on laminin-coated 12 mm glass coverslips (0.5 μg laminin/cm^2^ coating overnight at 4°C; Gibco rhLaminin-521) and allowed to adhere for up to 3 days. For membrane extraction (demembranation), adhered islets were washed three times in warm PBS (NaCl 137 mM, KCl 2.7 mM, Na_2_HPO_4_ 10 mM, KH_2_PO_4_ 1.8 mM, pH 7.4), then incubated for 5 min in cytoskeleton buffer (50 mM imidazole, 50 mM KCl, 0.5 mM MgCl_2_, 0.1 mM EDTA, 1 mM EGTA, pH 6.8) with 0.5% Triton X-100 and 0.25% glutaraldehyde, followed by 10 min in cytoskeleton buffer with 2% Triton X-100 and 1% CHAPS.

### Antibody staining and immuno-gold labeling

Demembranated islets were washed in PBS three times, then fixed with 4% PFA in PBS for 20 min at room temperature with gentle shaking. Post-fixation, islets were quenched with 50 mM glycine (Rosas-Arellano et al., 2016) in PBS for 15 min, and incubated for 30 min in blocking solution consisting of 1% BSA in PBS and 5% normal donkey serum (NDS) in PBS. Primary antibody labeling was performed at 4°C for 24 h and then room temperature for 5 h. Primary antibodies were used between 1:50–1:400 dilution in blocking solution; specific dilutions are indicated in Table 1. Negative controls were performed using secondary antibody alone. After primary antibody labeling, islets were washed five times for 5 min each in blocking solution. Secondary gold labeling was performed for 1.5 h at 1:20 dilution in blocking solution (18 nm gold donkey-anti-mouse- and donkey-anti-rabbit-IgG, Jackson ImmunoResearch, 705-215-147, 711-215-152). Post-staining, islets were washed twice for 5 min each in blocking solution, then three times for 5 min each in PBS, and fixed in 2% glutaraldehyde (GA) in PBS for 15 min.

### SEM

Coverslips containing adhered and fixed islets were stained with 0.5% osmium tetroxide in PBS for 20 min on ice. Samples were rinsed three times for 10 min each in ultrapure water and dehydrated in a graded ethanol series (10%, 20%, 30%, 50%, 70%, 90%, 100%, repeated three times) for 5 min in each step. Once dehydrated, samples were loaded into a critical point drier (Leica EM CPD 300, Vienna, Austria), which was set to perform 12 CO_2_ exchanges at the slowest speed and dried. Samples were then mounted on aluminum stubs with carbon adhesive tabs and coated with 12 nm of carbon (Leica ACE 600, Vienna, Austria). Finished samples were stored in a lab desiccator until imaging. SEM images were acquired on a Helios 5 UX DualBeam FIB-SEM platform (Fisher Scientific, Brno, Czech Republic) using SEM imaging mode at 5 kV and 0.1 nA using an Everhart–Thornley detector (ETD) for imaging both secondary and backscatter electrons in low magnification (1200×), and a through lens detector (TLD) and in-column detector (ICD) at higher magnifications (50,000–100,000×) to separately image secondary electron and backscatter electron signal, respectively.

## Supplementary Material


