# Supplementary material for: Immuno-scanning electron microscopy of islet primary cilia
Source: J Cell Sci. 2024 May 28;137(20):jcs262038. doi: 10.1242/jcs.262038 (PMC11166453; doi:10.1242/jcs.262038)
Supplement: Supplementary file 1 [file joces-137-262038_review_history.pdf]

Journal of Cell Science

Immuno-scanning electron microscopy of islet primary cilia

Sanja Sviben, Alexander J. Polino, Isabella L. Melena and Jing W. Hughes
DOI: 10.1242/jcs.262038

Editor: Lucy Collinson

Review timeline

Original submission:	22 February 2024
Editorial decision:	28 March 2024
First revision received:	15 April 2024
Accepted:	19 April 2024

Original submission

First decision letter

MS ID#: JOCES/2024/262038

MS TITLE: Immuno-scanning electron microscopy of islet primary cilia

AUTHORS: Sanja Sviben, Alexander J Polino, Isabella L Melena, and Jing W Hughes

ARTICLE TYPE: Research Article

We have now reached a decision on the above manuscript.

To see the reviewers' reports and a copy of this decision letter, please go to: https://submit-jcs.biologists.org and click on the 'Manuscripts with Decisions' queue in the Author Area. (Corresponding author only has access to reviews.)

As you will see, the reviewers gave favourable reports but raised some critical points that will require amendments to your manuscript. I hope that you will be able to carry these out because I would like to be able to accept your paper, depending on further comments from reviewers.

Please ensure that you clearly highlight all changes made in the revised manuscript. Please avoid using 'Tracked changes' in Word files as these are lost in PDF conversion.

I should be grateful if you would also provide a point-by-point response detailing how you have dealt with the points raised by the reviewers in the 'Response to Reviewers' box. Please attend to all of the reviewers' comments. If you do not agree with any of their criticisms or suggestions please explain clearly why this is so.

Reviewer 1

Advance summary and potential significance to field

Journal of Cell Science / Manuscript JOCES/2024/262038

Sanja Sviben, Alexander J. Polino, Isabella L. Melena, and Jing W. Hughes Immuno-scanning electron microscopy of islet primary cilia

© 2024. Published by The Company of Biologists under the terms of the Creative Commons Attribution License (https://creativecommons.org/licenses/by/4.0/).

Preliminary note: In my reviewer area of the JCS website the article type for the manuscript is listed as a research article. The manuscript is certainly not suitable for a research article, but perhaps this is just a mistake, as the manuscript would be perfectly suitable for a tools and resources article.

In the manuscript submitted by Sviben et al. the authors describe a method for immunogold labeling of primary cilia of isolated pancreatic islet cells and subsequent analysis of the samples by scanning electron microscopy (immuno-SEM). Immunogold labeling was tested on isolated mouse and human islet cells using seven different antibodies, all of which were successful except for one antibody, which showed very high background labeling (which is not uncommon). The description of the method is very detailed and understandable, so it should be no problem to reproduce it, even with samples other than isolated islet cells. The various parameters that might influence the intensity of the labeling and the ultrastructural preservation of the samples are discussed in detail. The authors, and I really like this, also talk about continuity of workflow (sensitivity of mouse islets to prolonged storage between critical point drying and coating) and imaging parameters. I find the use of 0.25 % glutaraldehyde as a fixative during the first step of demembranation particularly interesting. This apparently preserves axoneme-associated and juxta-membrane ciliary proteins (IFT88, Arl13b) on the axoneme at least in sufficient amounts to allow successful immunogold labeling. This is a very important point, as the protocol could easily be modified into a pre-embedding immunogold labeling protocol for transmission electron microscopy.
The study is of very high technical quality, the data are comprehensive, convincing, and well presented and I have no reservations about the importance and interest of the method described. The method will certainly make an important contribution to the field of cilia biology and as such this paper is likely to be a well-referenced paper for future studies in the field. As such the manuscript merits publication in the Journal of Cell Science. However, a few minor comments listed below should be addressed prior to publication.

Comments for the author

One point should be addressed:
The authors incubate their samples in the primary antibodies for 24 hours at 4°C and then for another 5 hours at room temperature. This is a very long incubation time. Is there a specific reason why such a long incubation time is really necessary? My own experience shows that long incubation times (overnight versus 1 – 2 hours) in PBS, which after all contains 150 mM NaCl, can significantly degrade the ultrastructure of the cilia. Even in the case of post-embedding immunogold labeling experiments with the cilia embedded in plastic (comparable fixation conditions, i.e. 0.25 % glutaraldehyde and 4 % formaldehyde). Could the authors please comment on the really long incubation time they do use for the primary antibodies?

Some minor points, typos etc:
Page 2, section “Antibody staining and immuno-gold labeling”, line 4: “…. consisting of 1% BSA (100 mg BSA in 10 mL PBS) ….”. The reference that 1% BSA is 100 mg BSA in 10 mL PBS certainly doesn't hurt anyone, but it’s not really necessary. Especially since this is the only place where such an example calculation is given. Just "1% BSA in PBS" is sufficient.

Page 6, section “Antibody performance”, line 10, 11: “…. used commercial 12 nm colloidal gold conjugated Ig to ….”. In the Introduction and Methods section the authors write that they used 18 nm colloidal gold conjugates.

Page 5, section “Sample stability and preservation”, last sentence and page 6, section “sample charging and coating”, line 6: At these points the authors refer to "grid" in connection with their samples, by which I would understand that the pancreatic islets are on a normal TEM grid. If I understand the procedure described in the Methods section correctly, the pancreatic islets are adhered onto coverslips. The authors should clarify this point.

Figures and figure legends: In the figures, the individual images are labeled with uppercase letters, while in the figure legends lowercase letters are used. This is somewhat unusual.

© 2024. Published by The Company of Biologists under the terms of the Creative Commons Attribution License (https://creativecommons.org/licenses/by/4.0/).

Figure 1, A: The authors should label some more primary cilia with an arrow. Especially in the upper right quadrant, there are a number of primary cilia that are at least as easy to see (at least on my screen) as the already marked cilia in the lower left quadrant.

Figure 1., Legend, (a): “An intact mouse pancreatic islet scanned at …. “. To be completely clear, the authors should write here “An intact, demembranated mouse pancreatic islet scanned at”.

Reviewer 2

Advance summary and potential significance to field

ImmunoSEM is used to label and localise proteins of the ciliary axoneme in islet cells. This paper is an excellent detailed description of a technique which clearly outlines the materials, methodology and possible pitfalls of immunoSEM. It provides enough detail to be reproduced. It is not a novel technique but this appears to tbe the first appilication to islet cells.
Comments below outline suggestions for improvement.

Comments for the author

Reviewer 3

Advance summary and potential significance to field

The manuscript by Sviben et al. “Immuno-Scanning Electron Microscopy of Islet Primary Cilia” describes the use of surface scanning electron microscopy (SEM) coupled with antibody-based labeling of specific ciliary proteins to determine cilia localization in pancreatic islands. The work therefore tackles a very important aspect in the study of cilia biology (and cell biology in general) which is bypassing the resolution limit of light microscopy in localization studies.

The study clearly shows the expertise of the group both from a technical point of view as well as in working with pancreatic cilia. The main advance presented here is the possibility of using SEM to assess the localization of proteins that are located inside the cilium which is achieved by a demembranation step coupled to other technical steps that are described in great detailed. The study thus shows SEM localization data for different ciliary proteins including axonemal components such as tubulin (both acetylated and glutamylated) and motor and IFT components. The quality of the results is simply outstanding.

Also, the authors do a great job providing protocol tips and guidelines that will surely benefit the work of other colleagues.

In sum, the work is solid and presents a novel technical approach to study protein localization in cilia with a level of detailed that undoubtedly will be instrumental to answer basic questions about cilia biology.

Finally, the work is presented as a Research article but is seems that it will fit perfectly in the journal as a Tools & Resource article. Having said that, I highly recommend publication of the study.

Comments for the author

One aspect that I believe will be very interesting and important is to discuss pros and cons, or requirements for using this technical approach to study cilia in other cell types, tissues or organs. A section in the discussion addressing the possible general use of the approach to study cilia in different contexts will surely be of great interest.

© 2024. Published by The Company of Biologists under the terms of the Creative Commons Attribution License (https://creativecommons.org/licenses/by/4.0/).

First revision

Author response to reviewers' comments

JOCES/2024/262038 **Response from authors:**

We are grateful to the editor and all three reviewers for their constructive feedback on our work. We have incorporated all reviewer suggestions into the revised manuscript. Point-by-point responses below.

Also, per Editorial Office request:
- since tracked changes were not allowed in the manuscript Word document, we highlighted all text changes in yellow.
- the original Figures 4-5 and 7-8 were consolidated into the new Figures 4 and 6, to fit within the allotted number of 9 total figures/tables.
- Methods section moved to after Discussion.
- in-text citations reformatted to JCS style, showing author name/year instead of numbers.
- high-resolution figure images provided as individual Jpeg files.

Reviewer 1 Advance Summary and Potential Significance to Field...

Journal of Cell Science / Manuscript JOCES/2024/262038

Sanja Sviben, Alexander J. Polino, Isabella L. Melena, and Jing W. Hughes
Immuno-scanning electron microscopy of islet primary cilia

Preliminary note: In my reviewer area of the JCS website the article type for the manuscript is listed as a research article. The manuscript is certainly not suitable for a research article, but perhaps this is just a mistake, as the manuscript would be perfectly suitable for a tools and resources article.

In the manuscript submitted by Sviben et al. the authors describe a method for immunogold labeling of primary cilia of isolated pancreatic islet cells and subsequent analysis of the samples by scanning electron microscopy (immuno-SEM). Immunogold labeling was tested on isolated mouse and human islet cells using seven different antibodies, all of which were successful except for one antibody, which showed very high background labeling (which is not uncommon). The description of the method is very detailed and understandable, so it should be no problem to reproduce it, even with samples other than isolated islet cells. The various parameters that might influence the intensity of the labeling and the ultrastructural preservation of the samples are discussed in detail. The authors, and I really like this, also talk about continuity of workflow (sensitivity of mouse islets to prolonged storage between critical point drying and coating) and imaging parameters. I find the use of 0.25 % glutaraldehyde as a fixative during the first step of demembranation particularly interesting. This apparently preserves axoneme-associated and juxta-membrane ciliary proteins (IFT88, Arl13b) on the axoneme at least in sufficient amounts to allow successful immunogold labeling. This is a very important point, as the protocol could easily be modified into a pre- embedding immunogold labeling protocol for transmission electron microscopy.
The study is of very high technical quality, the data are comprehensive, convincing, and well presented and I have no reservations about the importance and interest of the method described. The method will certainly make an important contribution to the field of cilia biology and as such this paper is likely to be a well-referenced paper for future studies in the field. As such the manuscript merits publication in the Journal of Cell Science. However, a few minor comments listed below should be addressed prior to publication.

We thank the reviewer for these favorable comments. Have re-listed this manuscript as a Tools and Resources article.

Reviewer 1 Comments for the Author...

© 2024. Published by The Company of Biologists under the terms of the Creative Commons Attribution License (https://creativecommons.org/licenses/by/4.0/).

One point should be addressed:
The authors incubate their samples in the primary antibodies for 24 hours at 4°C and then for another 5 hours at room temperature. This is a very long incubation time. Is there a specific reason why such a long incubation time is really necessary? My own experience shows that long incubation times (overnight versus 1 – 2 hours) in PBS, which after all contains 150 mM NaCl, can significantly degrade the ultrastructure of the cilia. Even in the case of post-embedding immunogold labeling experiments with the cilia embedded in plastic (comparable fixation conditions, i.e. 0.25 % glutaraldehyde and 4 % formaldehyde). Could the authors please comment on the really long incubation time they do use for the primary antibodies?

Thanks - this is an important point. We could have tested shorter incubation times and there is space for improvement here. Due to limited resources in particular human islets that were unavailable for additional testing, we relied on our experience with immunoSEM in other tissues to use longer incubation times. This approach worked well in our initial tests in islets, so we stuck to that protocol. We appreciate the reviewer's suggestion that shorter times may help minimize degradation. We have added this point to the Discussion and will certainly test this in future immunoSEM studies. Thanks once again for this helpful suggestion.

Some minor points, typos etc:
Page 2, section "Antibody staining and immuno-gold labeling", line 4: "…. consisting of 1% BSA (100 mg BSA in 10 mL PBS) ….". The reference that 1% BSA is 100 mg BSA in 10 mL PBS certainly doesn't hurt anyone, but it's not really necessary. Especially since this is the only place where such an example calculation is given. Just "1% BSA in PBS" is sufficient.
Thanks, corrected.

Page 6, section "Antibody performance", line 10, 11: "…. used commercial 12 nm colloidal gold conjugated Ig to ….". In the Introduction and Methods section the authors write that they used 18 nm colloidal gold conjugates.
Thanks, 18 nm is correct.

Page 5, section "Sample stability and preservation", last sentence and page 6, section "sample charging and coating", line 6: At these points the authors refer to "grid" in connection with their samples, by which I would understand that the pancreatic islets are on a normal TEM grid. If I understand the procedure described in the Methods section correctly, the pancreatic islets are adhered onto coverslips. The authors should clarify this point.
Coverslip is the correct terminology. We have updated this in both places.

Figures and figure legends: In the figures, the individual images are labeled with uppercase letters, while in the figure legends lowercase letters are used. This is somewhat unusual.
Uppercase is now used throughout, thanks for pointing this out.

Figure 1, A: The authors should label some more primary cilia with an arrow. Especially in the upper right quadrant, there are a number of primary cilia that are at least as easy to see (at least on my screen) as the already marked cilia in the lower left quadrant.
We have updated Figure 1A to include more arrows.

Figure 1., Legend, (a): "An intact mouse pancreatic islet scanned at …. ". To be completely clear, the authors should write here "An intact, demembranated mouse pancreatic islet scanned at".
Revised as suggested, thanks.

Reviewer 2 Advance Summary and Potential Significance to Field...

ImmunoSEM is used to label and localise proteins of the ciliary axoneme in islet cells. This paper is an excellent detailed description of a technique which clearly outlines the materials, methodology and possible pitfalls of immunoSEM. It provides enough detail to be reproduced. It is not a novel technique but this appears to tbe the first appilication to islet cells.
Comments below outline suggestions for improvement.

Reviewer 2 Comments for the Author...

© 2024. Published by The Company of Biologists under the terms of the Creative Commons Attribution License (https://creativecommons.org/licenses/by/4.0/).

[View uploaded comments for the author]

Thank you - hugely appreciate all the critical comments and careful proof editing. Have addressed each point in the PDF and made corresponding revisions in the manuscript. Thanks very much.

Reviewer 3 Advance Summary and Potential Significance to Field...

The manuscript by Sviben et al. "Immuno-Scanning Electron Microscopy of Islet Primary Cilia" describes the use of surface scanning electron microscopy (SEM) coupled with antibody-based labeling of specific ciliary proteins to determine cilia localization in pancreatic islands. The work therefore tackles a very important aspect in the study of cilia biology (and cell biology in general) which is bypassing the resolution limit of light microscopy in localization studies.

The study clearly shows the expertise of the group both from a technical point of view as well as in working with pancreatic cilia. The main advance presented here is the possibility of using SEM to assess the localization of proteins that are located inside the cilium which is achieved by a demembranation step coupled to other technical steps that are described in great detailed. The study thus shows SEM localization data for different ciliary proteins including axonemal components such as tubulin (both acetylated and glutamylated) and motor and IFT components. The quality of the results is simply outstanding.

Also, the authors do a great job providing protocol tips and guidelines that will surely benefit the work of other colleagues.

In sum, the work is solid and presents a novel technical approach to study protein localization in cilia with a level of detailed that undoubtedly will be instrumental to answer basic questions about cilia biology.

Finally, the work is presented as a Research article but is seems that it will fit perfectly in the journal as a Tools & Resource article. Having said that, I highly recommend publication of the study.

Reviewer 3 Comments for the Author...

One aspect that I believe will be very interesting and important is to discuss pros and cons, or requirements, for using this technical approach to study cilia in other cell types, tissues or organs. A section in the discussion addressing the possible general use of the approach to study cilia in different contexts will surely be of great interest.

Great suggestion - we added a penultimate paragraph on this:

"Based on the reproducibility of immunogold labeling between mouse and human islets and among sample preparations, we believe our immuno-SEM protocol to be potentially applicable to studying cilia in broader contexts, including primary cilia in non-islet cells, motile cilia in multi-ciliated cells, embryonic nodal cilia, stereocilia, and indeed any other surface organelle. The basic requirements for feasibility of this approach include: a) the structure of interest is stably exposed on the cell surface or otherwise accessible by SEM, b) the specimen can be affixed to an imaging surface e.g. glass coverslip and survive extensive fixation and washing steps, and c) validated primary antibodies exist for the proteins of interest. While we only performed single protein labeling here, multiplexed labeling can be done using different sizes of gold particles e.g. 12 and 18 nm. Demembranation may help expose intracellular epitopes on the core axoneme, while transmembrane proteins may be better detected on native, membrane-intact specimens."

Thanks to all reviewers for helping to improve our manuscript.

Second decision letter

© 2024. Published by The Company of Biologists under the terms of the Creative Commons Attribution License (https://creativecommons.org/licenses/by/4.0/).

MS ID#: JOCES/2024/262038

MS TITLE: Immuno-scanning electron microscopy of islet primary cilia

AUTHORS: Sanja Sviben, Alexander J Polino, Isabella L Melena, and Jing W Hughes

I am happy to tell you that your manuscript has been accepted for publication in Journal of Cell Science, pending standard ethics checks.

© 2024. Published by The Company of Biologists under the terms of the Creative Commons Attribution License (https://creativecommons.org/licenses/by/4.0/).
